# Effects of liquid feeding of corn condensed distiller’s solubles and whole stillage on growth performance, carcass characteristics, and sensory traits of pigs

**DOI:** 10.1186/s40104-016-0140-6

**Published:** 2017-01-19

**Authors:** Xiaojian Yang, Carissa Nath, Alan Doering, John Goihl, Samuel Kofi Baidoo

**Affiliations:** 1 Southern Research and Outreach Center, University of Minnesota, Waseca, MN 56093 USA; 2 Agricultural Utilization and Research Institute, Marshall, MN 56258 USA; 3 Agricultural Utilization and Research Institute, Waseca, MN 56093 USA; 4 Agri-Nutrition Services, Inc., Shakopee, MN 55379 USA; 5 Department of Animal Science, University of Minnesota, St. Paul, MN 55108 USA

**Keywords:** Carcass, Condensed distiller’s solubles, Liquid feeding, Performance, Pig, Whole stillage

## Abstract

**Background:**

The immense growth in global bioethanol production has greatly increased the supply of by-products such as whole stillage and condensed distiller’s solubles, which could be potentially used for animal feeding. The objective of this study was to investigate effects of liquid feeding high levels of corn condensed distiller’s solubles (CCDS) and whole stillage (CWS) on growth performance, carcass characteristics, belly firmness and meat sensory traits of pigs.

**Methods:**

A total of 256 pigs were blocked by sex and initial BW (13.5 ± 2.5 kg), and pens of pigs (8 pigs/pen) were randomly allocated to 1 of 4 dietary treatments (8 pens/treatment): 1) corn-soybean meal based diet as control, 2) 25% CWS + 5% CCDS, 3) 19.5% CWS + 10.5% CCDS, and 4) 19.5, 26, and 32.5% CWS + 10.5, 14, and 17.5% CCDS in phases 1 (28 d), 2 (38 d), and 3 (60 d), respectively. Inclusion levels of CCDS and CWS for Treatments 1, 2, and 3 were fixed during all the three phases of the experiment. Inclusion levels of CWS and CCDS were on 88% dry matter basis. The liquid feeding system delivered feed from the mixing tank to feed troughs by high-pressure air, had sensors inside feed troughs, and recorded daily feed intake on the basis of a reference feed intake curve. The pigs were fed 5 to 10 times per day with increasing frequency during the experiment.

**Results:**

Control pigs had greater (*P* < 0.05) average daily gain (0.91 vs. 0.84, 0.85, 0.85 kg/d) and gain to feed ratio (0.37 vs. 0.33, 0.34, 0.34) than pigs in the other three treatments during the overall period. Compared with the control, the other three groups had (*P* < 0.05) or tended to have (*P* < 0.10) lower carcass weight and backfat depth due to lighter (*P* < 0.05) slaughter body weight, but similar (*P* > 0.10) dressing percentage, loin muscle depth, and lean percentage were observed among the four treatments. Inclusion of CWS and CCDS reduced (*P* < 0.05) or tended to reduce (*P* < 0.10) belly firmness but did not influence (*P* > 0.10) the overall like, flavor, tenderness and juiciness of loin chops when compared with the control group.

**Conclusion:**

In conclusion, our results indicate that including 30–50% of a mixture of whole stillage and condensed distiller’s solubles in the growing-finishing diets may reduce growth performance, carcass weight and belly firmness, but does not affect pork sensory traits.

## Background

The U.S. ethanol production has risen tremendously from near 660 million liters in 1980 to 54 billion liters in 2014 [1]. Corn is the main raw material, which can be converted into ethanol by either the dry grind or wet milling process. The dry grind technique needs less capital investment and energy consumption and represents the method adopted in the majority of the ethanol plants in the U.S. [2]. In the dry grind process of ethanol production, the whole corn kernel is ground into flour and mixed with water to form a mash. The mash is cooked in a high-temperature cooker to decrease levels of bacteria and enzymes (e.g. alpha amylase and glucoamylase) are added to convert starch to dextrose. Then yeast is added to ferment the sugars and the resulting mixture is transferred to distillation columns where the ethanol is separated from the remaining stillage. The stillage is called whole stillage which contains about 10% dry matter and can be further centrifuged or screened to produce wet distiller’s grains and thin stillage. Through the condensation process, thin stillage is concentrated by evaporation to condensed distiller’s solubles containing about 30% dry matter. Recently, the interest in utilization of ethanol by-products containing relative high moisture as livestock feed ingredients has increased due to the increased availability of the co-products and high cost associated with drying. Corn condensed distiller’s solubles (CCDS) and whole stillage (CWS) are high moisture ethanol by-products that may be potentially used for animal feeding. However, research on liquid feeding of swine with inclusion of high moisture ethanol by-products is limited. As reviewed by Lane [3], research in the late 1940’s indicated that feeding whole stillage alone to pigs caused soft carcass, yet the carcass quality was improved if dry corn was added to the diet. Squire et al. [4] reported that liquid feeding CCDS at 15% of diet dry matter did not affect finishing pig carcass characteristics, but slightly reduced growth performance of growing pigs when compared with the corn-soybean meal control. In addition, they observed a decrease in feed intake if the level of CCDS was increased to 22.5% [4]. It has been shown that inclusion of up to 30% corn distiller’s dried grains with solubles (DDGS) in growing-finishing pig diets might not adversely impact pig performance [5], but further increase of DDGS inclusion level to 45% could reduce growth performance of pigs [6]. Lee et al. [7] reported that, on a dry matter basis, CWS consisted of about 80% of distiller’s grains and 20% of stillage solubles. Hence, including CWS at 20% of diet dry matter approximately contributes to 16% of distiller’s grains and 4% of stillage solubles in the diet. If CCDS is also included in this diet at 5% on a dry matter basis, the total amount of stillage solubles is 9% in the diet. Similarly, on a dry matter basis, inclusion of 25% of CWS provides 20% of distiller’s grains and 5% of stillage solubles in the diet. With addition of 10% CCDS, the total amount of stillage solubles in the diet is 15%. If a diet contains, on a dry matter basis, 30% of CWS and 20% of CCDS, the total amount of distiller’s grains is 24% and level of stillage solubles is 26% in the diet. We hypothesized that including CCDS and CWS with their total level at 30% of diet dry matter would not negatively influence performance of growing-finishing pigs, and that further increasing their total level to 50% would impair pig growth performance. Moreover, settling, a process by which particulates settle to the bottom of a liquid, is a concern when CWS is used in liquid feeding and including high viscosity CCDS may help reduce feed separation [8]. For example, Lu and Rosentrater [9] observed that settling happened in whole stillage after 1 h of storage. Therefore, the objective of our study was to investigate effects of liquid feeding high levels of mixture of CWS and CCDS on growth performance, carcass characteristics, belly firmness and meat sensory traits of pigs.

## Methods

The experimental protocol was reviewed and approved by the University of Minnesota Institutional Animal Care and Use Committee. The experiment was conducted at the Southern Research and Outreach Center, University of Minnesota, in Waseca, MN, USA.

### Nutrient composition of feed ingredients

Contents of moisture, crude protein, crude fat, neutral detergent fiber (NDF), acid detergent fiber (ADF), ash, calcium, phosphorus, sulfur, and amino acids in feed ingredients used in this study including corn, soybean meal, corn condensed distiller’s solubles (CCDS) and whole stillage (CWS) were analyzed before the trial was started. The ethanol by-products CCDS and CWS were freshly obtained from a nearby ethanol plant (Guardian Energy, LLC; Janesville, MN, USA). Dry grind process is used for production of ethanol in this plant. The nutrient composition of the two by-products is presented in Table 1. During the initial phase of the feeding experiment, one batch of fresh CCDS and CWS were obtained approximately every 10 d and 5 d, respectively. Gradually the co-products were picked up more frequently. In later phase of the trial, one batch of fresh CCDS and CWS were used approximately every 5 to 7 d and 2 to 3 d, respectively. All co-products were stored in tanks that were installed indoors and the room temperature was kept at about 10 (Winter) to 20 (Fall) °C. No preservatives were added to the ethanol co-products during the experimental period. Lu and Rosentrater [9] reported that in the absence of preservatives no mold was noticed in CCDS stored up to 10 d at a temperature of 12, 22, or 35 °C, and mold appeared in CWS on the 5^th^ and 8^th^ day of storage at a temperature of 22 and 12 °C, respectively.

**Table 1:** Analyzed nutrient composition of the by-products, as-fed basis

	Corn condensed distiller’s solubles (CCDS)	Corn whole stillage (CWS)
Nutrients, %		
Moisture	68.51	87.20
Crude protein	8.11	3.70
Crude fat (acid hydrolysis)	1.86	2.32
Acid detergent fiber	0.23	1.89
Neutral detergent fiber	0.32	3.37
Ash	4.17	0.71
Calcium	0.01	0.0028
Phosphorus	0.75	0.14
Sulfur	0.58	0.06
Indispensable amino acids, %		
Arg	0.44	0.17
His	0.26	0.10
Ile	0.30	0.15
Leu	0.67	0.44
Lys	0.35	0.13
Met	0.14	0.07
Phe	0.33	0.19
Thr	0.32	0.14
Trp	0.07	0.03
Val	0.42	0.19
Dispensable amino acids, %		
Ala	0.46	0.25
Asp	0.48	0.23
Cys	0.17	0.08
Glu	0.66	0.46
Gly	0.38	0.14
Pro	0.58	0.29
Ser	0.36	0.17
Tyr	0.25	0.14

For diet formulation, metabolizable energy (ME) of CWS was assumed to be 3432 kcal/kg (88% dry matter basis), similar to the ME of corn dried distiller’s grains with solubles (DDGS) with >10% oil as listed in NRC [10]. The ME of CCDS (3012 kcal/kg on 88% dry matter basis), corn (3392 kcal/kg), soybean meal (3377 kcal/kg), and choice white grease (8118 kcal/kg) were based on NRC [10, 11]. The standardized ileal digestibility (SID) of amino acids for CWS were assumed to be the same as those in NRC [10] for DDGS with >10% oil, whereas the SID of amino acids in CCDS were based on reported values by Soares et al [12]. The SID of amino acids in corn and soybean meal were assumed to be the same as those in NRC [10]. Apparent total tract digestibility of phosphorus in corn (20%), soybean meal (31%), CCDS (60%), and CWS (60%) were assumed to be similar to the values listed in NRC [10] for the corresponding ingredients or DDGS.

### Experimental design and animal management

The pigs used in the study were offspring of dam Topigs 20 (Large White × Danish Landrace) and sire Duroc (Compart’s, Nicollet, MN, USA). A total of 256 crossbred pigs with an initial body weight 13.52 ± 2.54 kg were used in the present experiment. Pigs were blocked by body weight and sex and randomly assigned to 1 of 4 dietary treatments: 1) corn-soybean meal based diet (control), 2) 5% CCDS + 25% CWS, 3) 10.5% CCDS + 19.5% CWS, and 4) 10.5% CCDS + 19.5% CWS, 14% CCDS + 26% CWS, 17.5% CCDS + 32.5% CWS in phases 1 (28 d), 2 (38 d), and 3 (60 d), respectively. All percentages were on 88% dry matter basis. The ingredient and nutrient compositions of base mixes and diets are shown in Tables 2 and 3, respectively. Diets were formulated to meet or exceed estimates of requirements from NRC [10] and contain, on 88% dry matter basis, similar levels of ME and standardized ileal digestible (SID) lysine, and methionine, threonine, and tryptophan among the four treatments. Pigs were fed phases 1, 2, and 3 diets for 28, 38, and 60 d, respectively. The length of each phase was selected on the basis of pig body weight which was monitored during the experiment. At the end of each phase, the average body weight was approximately 35, 80, and 120 kg, respectively.

**Table 2:** Ingredient and nutrient composition of base mixes, as-fed basis

Item	Phase 1				Phase 2				Phase 3			
Treatment^a^	1	2	3	4	1	2	3	4	1	2	3	4
Ingredient, %												
Corn	60.32	58.64	58.08	58.08	66.58	67.87	67.39	67.89	71.02	73.11	72.54	68.02
Soybean meal	35.00	34.64	34.64	34.64	29.00	25.71	25.71	23.33	25.00	21.43	21.43	22.00
Choice white grease	1.00	1.57	2.20	2.20	1.30	2.03	2.61	3.57	1.12	1.64	2.26	4.28
Limestone	0.55	2.04	2.29	2.29	0.48	1.96	2.19	2.67	0.58	2.07	2.14	3.40
Dicalcium phosphate	1.79	0.86	0.50	0.50	1.50	0.43	0.07		1.30	0.14		
Lysine HCl	0.28	0.67	0.67	0.67	0.22	0.60	0.59	0.82	0.15	0.46	0.46	0.68
DL-Methionine	0.17	0.21	0.23	0.23	0.08	0.10	0.11	0.13	0.03	0.01	0.03	
L-Threonine	0.09	0.17	0.19	0.19	0.04	0.10	0.13	0.18				
L-Tryptophan		0.06	0.06	0.06		0.06	0.06	0.08				0.02
Sodium chloride	0.30	0.43	0.43	0.43	0.30	0.43	0.43	0.50	0.30	0.43	0.43	0.60
Mineral-vitamin premix^b^	0.50	0.71	0.71	0.71	0.50	0.71	0.71	0.83	0.50	0.71	0.71	1.00
Nutrients^c^, %												
Dry matter^d^	89.70	90.11	90.19	90.19	89.53	89.71	89.70	90.14	87.81	87.93	88.00	88.62
GE, kcal/kg^d^	3867	3889	3935	3935	3896	3908	3917	3947	3735	3774	3781	3853
ME, kcal/kg	3344	3344	3368	3368	3368	3392	3415	3439	3368	3368	3392	3439
Crude fat^d^	3.51	4.43	4.59	4.59	3.58	4.25	4.31	6.25	3.23	3.61	3.63	5.68
Crude protein^e^	21.38	21.58	21.57	21.57	18.88	17.89	17.87	17.07	17.20	15.96	15.93	16.07
	(19.30)	(20.65)	(21.66)	(21.66)	(18.75)	(17.28)	(17.61)	(18.56)	(18.21)	(15.59)	(16.13)	(16.56)
Calcium	0.80	1.14	1.15	1.15	0.68	0.97	0.97	1.13	0.65	0.92	0.92	1.40
Total phosphorus	0.65	0.48	0.41	0.41	0.57	0.36	0.29	0.26	0.52	0.29	0.26	0.26
ATTD phosphorus^f^	0.36	0.22	0.16	0.16	0.30	0.14	0.08	0.07	0.27	0.09	0.07	0.07
Total Lys	1.38	1.67	1.67	1.67	1.17	1.37	1.36	1.47	1.01	1.15	1.15	1.32
SID Lys^g^	1.25	1.54	1.54	1.54	1.05	1.26	1.26	1.37	0.90	1.05	1.05	1.23

^a^Treatment 1, corn-soybean meal diet; Treatment 2, 5% CCDS + 25% CWS, Treatment 3, 10.5% CCDS + 19.5% CWS; Treatment 4, graded level CCDS + graded level CWS

^b^The vitamin and trace mineral premix provided the following (per kg of final diets listed in Table 3): vitamin A, 11,000 IU; vitamin D_3_, 2756 IU; vitamin E, 55 IU; vitamin B_12_, 55 μg; riboflavin, 16,000 mg; pantothenic acid, 44.1 mg; niacin, 82.7 mg; Zn, 150 mg; Fe, 175 mg; Mn, 60 mg; Cu, 17.5 mg; I, 2 mg; and Se, 0.3 mg

^c^Calculated values, unless stated otherwise

^d^Analyzed values

^e^Analyzed values in brackets

^f^ *ATTD* apparent total tract digestible

^g^ *SID* standardized ileal digestible

**Table 3:** Ingredient composition and calculated nutrient composition of diets, adjusted to 88% dry matter basis^a^

Item	Phase 1				Phase 2				Phase 3			
Dietary treatment^b^	1	2	3	4	1	2	3	4	1	2	3	4
Ingredient, %												
Base mixes	100.0	70.0	70.0	70.0	100.0	70.0	70.0	60.0	100.0	70.0	70.0	50.0
CCDS		5.0	10.5	10.5		5.0	10.5	14.0		5.0	10.5	17.5
CWS		25.0	19.5	19.5		25.0	19.5	26.0		25.0	19.5	32.5
Nutrient content^c^, %												
ME, kcal/kg	3272	3296	3296	3296	3296	3320	3320	3344	3368	3368	3368	3344
Crude protein^d^	20.97 (19.83)	22.25 (21.51)	22.07 (21.90)	22.07 (22.10)	18.55 (19.13)	19.78 (20.16)	19.61 (20.25)	19.79 (19.88)	17.24 (18.19)	18.67 (18.95)	18.49 (16.90)	20.21 (17.83)
Calcium	0.78	0.78	0.79	0.79	0.67	0.67	0.67	0.66	0.65	0.65	0.65	0.70
Total phosphorus	0.64	0.67	0.69	0.69	0.56	0.59	0.61	0.70	0.52	0.55	0.59	0.81
ATTD phosphorus	0.35	0.35	0.35	0.35	0.30	0.30	0.30	0.37	0.27	0.27	0.29	0.44
Total Lys^d^	1.35 (1.30)	1.41 (1.35)	1.42 (1.28)	1.42 (1.32)	1.15 (1.21)	1.21 (1.13)	1.21 (1.25)	1.23 (1.14)	1.01 (0.95)	1.08 (1.09)	1.08 (0.98)	1.12 (1.10)
Total Met^d^	0.48 (0.46)	0.50 (0.48)	0.51 (0.49)	0.51 (0.50)	0.36 (0.37)	0.39 (0.36)	0.40 (0.38)	0.41 (0.39)	0.29 (0.32)	0.32 (0.35)	0.32 (0.32)	0.35 (0.35)
Total Met + Cys^d^	0.81 (0.79)	0.89 (0.91)	0.89 (0.89)	0.89 (0.88)	0.66 (0.65)	0.75 (0.72)	0.75 (0.70)	0.78 (0.75)	0.57 (0.64)	0.66 (0.70)	0.66 (0.64)	0.74 (0.72)
Total Thr^d^	0.88 (0.89)	0.95 (0.90)	0.95 (0.89)	0.95 (0.91)	0.73 (0.74)	0.80 (0.83)	0.82 (0.80)	0.84 (0.81)	0.63 (0.71)	0.69 (0.79)	0.68 (0.70)	0.75 (0.79)
Total Trp^d^	0.28 (0.27)	0.29 (0.28)	0.29 (0.28)	0.29 (0.27)	0.24 (0.23)	0.25 (0.24)	0.25 (0.23)	0.25 (0.24)	0.21 (0.21)	0.19 (0.22)	0.19 (0.20)	0.21 (0.23)
SID Lys	1.23	1.22	1.22	1.22	1.04	1.04	1.03	1.03	0.90	0.90	0.91	0.89
SID Met	0.45	0.45	0.45	0.45	0.34	0.34	0.34	0.34	0.27	0.27	0.27	0.27
SID Met + Cys	0.74	0.75	0.74	0.74	0.60	0.62	0.61	0.61	0.51	0.53	0.52	0.54
SID Thr	0.78	0.78	0.78	0.78	0.65	0.65	0.65	0.65	0.55	0.54	0.53	0.54
SID Trp	0.25	0.25	0.25	0.25	0.21	0.21	0.21	0.21	0.19	0.16	0.16	0.16

^a^ *CCDS* corn condensed distiller’s solubles, *CWS* corn whole stillage, *ATTD* apparent total tract digestible, *SID* standardized ileal digestible

^b^Treatment 1, corn-soybean meal diet; Treatment 2, 5% CCDS + 25% CWS, Treatment 3, 10.5% CCDS + 19.5% CWS; Treatment 4, graded level CCDS + graded level CWS

^c^Calculated values, unless stated otherwise

^d^Analyzed values in brackets

Each treatment had eight replicate pens with eight pigs (four barrows and four gilts) per pen and 0.95 m^2^ per pig. Each pen had concrete slatted floors, a single liquid feeding trough (101 cm length × 28 cm width) fitted with a sensor inside the trough, a water nipple, and a water meter. Pigs were fed by a Big Dutchman liquid feeding system (Big Dutchman, Inc.; Calvesage, Germany), which was computer controlled and recorded daily feed intake that was automatically adjusted, based on a reference feed intake curve. The pigs were fed 5 to 10 times per day with frequency increasing during the trial. The mixing tank and pipelines were emptied and cleaned between feedings of each diet to avoid cross-contamination between treatments. Each time before preparation of a new batch of feed, the feed levels in all troughs were checked by the sensors inside the feed troughs. If the trough was empty, new feed would be supplied for that trough; otherwise, no new feed would be supplied for that trough for that specific feeding time. Dosing of base mixes, CCDS, CWS, and water into the mixing tank which was placed on load cells was monitored by the computer. After mixing in the mixing tank for 7 min, the mixed feed was delivered to individual feed troughs by high-pressure air. The two ethanol by-products, CCDS and CWS, were stored in two separate tanks and re-circulated each time before being metered in proportion to dry base mixes with the aim to reduce settling. Dry matter content of the complete liquid feed mixture was maintained between 27 and 31% and kept similar among treatments except that, in phase 3, dry matter content in Treatment 4 was about three percentage units lower than the other treatments due to the high inclusion levels of CCDS and CWS. Animal rooms had a negative pressure ventilation system. Room temperatures were gradually decreased from 26 °C in the beginning of the trial to 18 °C at the end of the experiment.

On d 1, 28, 84, and 126, all pigs were individually weighed and feed intake per pen recorded for calculation of average daily gain (ADG), feed intake (ADFI), and feed efficiency during the periods of d 1–28, 28–84, 84–126, and 1–126. Fresh samples of CCDS and CWS were taken roughly once a month for moisture analysis. In phase 3, diet samples were taken from each of the 32 feed troughs. ADFI was presented on 88% dry matter basis. Water disappearance from the nipple drinker in each pen was recorded by a water meter with the value of the smallest division being 0.4 L. Water consumed with feed dry matter was also recorded for calculation of total daily water disappearance during the whole experimental period.

### Measurement of belly firmness

In the last week of the trial, eight pigs per treatment (1 pig per pen), with similar barrow to gilt ratio and average body weight around 118 kg, were selected and sent to a local packer for evaluation of belly firmness according to the method described by Whitney et al. [13]. Briefly, belly length on a flat surface was measured and then it was placed on the sharp edge of a triangular stainless steel smoke stick. The distance between the two ends of the suspended belly was then measured for calculation of the angle, an indicator of belly firmness.

### Evaluation of loin sensory quality

Loin samples were collected from the same pigs chosen for belly firmness measurement as described above. Boneless center-cut loins were removed from the right side of each carcass and processed according to the Institutional Meat Purchasing Specifications [14]. These boneless center-cut loins were then transported to the meat lab of Minnesota’s Agricultural Utilization Research Institute for subsequent sensory evaluation. Consumer taste panels were conducted according to guidelines set forth by the American Meat Science Association [15]. Panelists were recruited from Marshall, Minnesota, USA. All consumers were 18 years of age or older and consumed pork on a regular basis. Seventy-one consumers participated in the study over eight different panel sessions. Prior to the taste panel sessions, chops were thawed for 48 h at 4 °C. On the appropriate day and time, chops were cooked on clamshell grills to a target internal temperature of 71 °C. Chops were cut into 1.27 cm × 1.27 cm × 2.54 cm cubes and placed in glass bowls. These glass bowls were covered in aluminum foil and then placed in a 44 °C warming oven until served. Panels were conducted in booths preventing panelist interaction. Each sample was coded with a random 3-digit number. All samples were served under red lights to limit differences in visual color. Panelists were instructed to rate pork chops for overall liking and liking for flavor, tenderness and juiciness on a 9-point hedonic scale (1 = dislike extremely, 9 = like extremely). Each panelist evaluated samples of all four treatments.

### Evaluation of carcass characteristics

To evaluate carcass characteristics including hot carcass weight, carcass yield, fat depth, loin depth, and percentage of lean at the end of the trial, pigs were transported to a commercial pork packing plant (Tyson Foods; Waterloo, IA, USA), where they were slaughtered and carcass measured with the Animal Ultrasound System (Animal Ultrasound Services, Ithaca, NY, USA) by placing the ultrasound parallel to the midline of the carcass for measurement of the average fat and loin depth spanning the last rib to the tenth rib and for prediction of lean with hot carcass weight, fat and loin depth.

### Chemical analysis

AOAC methods [16] were used for analysis of moisture (method 934.01), crude protein (method 984.13), crude fat by acid hydrolysis (method 954.02), acid detergent fiber (ADF; method 973.18), ash (method 942.05), calcium (method 975.03B), phosphorus (method 966.01), and sulfur (method 956.01) in feed ingredients, base mixes or diets sampled from feed troughs. A heat-stable alpha-amylase was utilized for analysis of neutral detergent fiber (NDF) using the Ankom^2000^ Fiber Analyzer (Ankom Technology, Macedon, NY, USA) according to the method of Van Soest et al. [17]. Amino acid composition in feed ingredients was determined according to the AOAC method 982.30 E (a, b, c) [16] by the University of Missouri Agricultural Experiment Station Chemical Laboratories in Columbia, MO, USA. Gross energy (GE) was measured using an adiabatic bomb calorimeter (IKA Works, Inc., NC, USA) with benzoic acid as a calibration standard.

### Statistical analysis

All data were analyzed using the MIXED procedure of SAS. Pen was the experimental unit for all responses. Growth performance data for the three phases were analyzed with repeated measures in time and the first order autoregressive covariance structure. Treatment, time, and interaction between treatment and time were included as fixed effects, and pen was considered as a random effect. The PLM procedure and the slice option of the MIXED procedure were used for multiple comparisons of means within different phases [18]. Overall (d 1–126) growth performance (ADG, ADFI, and GF), water disappearance, and carcass characteristics data were analyzed by analysis of variance, with treatment as a fixed effect. Belly firmness data were subjected to analysis of covariance with belly thickness as a covariate. Sensory quality was analyzed by mixed model with dietary treatment as a fixed effect and panelist as a random effect. Tukey test was employed for multiple comparisons in all analyses. Statistical significance level was set at 0.05 and probabilities between 0.05 and 0.10 were considered as a tendency. The least squares mean and standard error of the mean (SEM) were presented unless otherwise indicated.

## Results

### Growth performance

Crude protein was used as an indicator to evaluate how uniformly the feed was mixed and delivered to the feed troughs in phase 3. The coefficient of variation of crude protein for troughs within a treatment was 15.5, 7.5, 10.0, and 13.7% for Treatments 1, 2, 3, and 4, respectively. The average coefficient of variation for the four treatments was 11.7%.

Two pigs (1 pig from Treatments 1 and 2, respectively) were removed during the experiment for reasons unrelated to the dietary treatments. Growth performance is shown in Table 4. No difference (*P* > 0.05) was observed for body weight across treatments on d 28. But on d 84 and 126, pigs fed diets containing CCDS and CWS (Treatments 2, 3, and 4) were lighter (*P* < 0.05) than pigs fed the control diet. Body gain followed a similar pattern to that of body weight. No difference (*P* > 0.05) in ADG was noticed during the period of d 1–28. Pigs in the control group gained more (*P* < 0.05) than the other three groups from d 28 to 84. However, from d 84 to 126, only pigs fed the diet containing 5% CCDS + 25% CWS (Treatment 2) had lower (*P* < 0.05) ADG than pigs fed the control diet. Overall, inclusion of CCDS and CWS (Treatments 2, 3, and 4) led to a reduction (*P* < 0.05) of ADG in comparison with the corn-soybean meal based control diet.

**Table 4:** Effects of liquid feeding ethanol byproducts on growth performance and water consumption of pigs^1,^ ^2^

Item	Dietary treatment					
	Treatment 1 (corn-soybean meal)	Treatment 2 (5% CCDS + 25% CWS)	Treatment 3 (10.5% CCDS + 19.5% CWS)	Treatment 4 (graded level CCDS + graded level CWS)	SEM^3^	*P* value
Body weight, kg						
d 1	13.4	13.6	13.6	13.5	0.7	1.00
d 28	34.8	34.7	34.8	34.2	1.1	0.984
d 84	84.8 ^A^	80.0 ^B^	79.9 ^B^	78.8 ^B^	1.5	0.010
d 126	127.7 ^A^	119.9 ^B^	120.8 ^B^	120.5 ^B^	1.6	0.0002
Average daily gain, kg/d						
d 1–28	0.76	0.75	0.76	0.74	0.02	0.791
d 28–84	0.89 ^A^	0.81 ^B^	0.81 ^B^	0.80 ^B^	0.02	0.004
d 84–126	1.02 ^A^	0.95 ^B^	0.97 ^AB^	0.99 ^AB^	0.01	0.02
d 1–126	0.91 ^A^	0.84 ^B^	0.85 ^B^	0.85 ^B^	0.01	0.003
Average daily feed intake (kg/d, 88% dry matter basis)						
d 1–28	1.15 ^Bb^	1.28 ^A^	1.27 ^A^	1.25 ^ABa^	0.04	0.008
d 28–84	2.23	2.32	2.25	2.26	0.03	0.176
d 84–126	3.70	3.74	3.65	3.66	0.001	0.113
d 1–126	2.48 ^B^	2.56 ^Aa^	2.50 ^ABb^	2.50 ^ABb^	0.02	0.014
Feed conversion efficiency (weight gain : feed intake)						
d 1–28	0.67 ^A^	0.59 ^B^	0.60 ^B^	0.59 ^B^	0.01	<0.0001
d 28–84	0.40 ^A^	0.35 ^B^	0.36 ^B^	0.35 ^B^	0.01	<0.0001
d 84–126	0.28	0.25	0.27	0.27	0.004	0.225
d 1–126	0.37 ^A^	0.33 ^B^	0.34 ^B^	0.34 ^B^	0.004	< 0.0001
Water disappearance during d 1–126 (l/pig⋅d)						
Water from feed	5.6 ^C^	5.8 ^B^	5.6 ^C^	6.3 A	0.03	< 0.0001
Water from drinkers	2.0	1.3	1.5	1.8	0.3	0.391
Total water	7.6 ^ab^	7.1 ^b^	7.1 ^b^	8.1 ^a^	0.3	0.056

^1^ *CCDS* corn condensed distiller’s solubles, *CWS* corn whole stillage

^2^Means within a row without common upper case letters differ (*P* < 0.05). Means within a row without common lower case letters tend to differ (0.05 < *P* < 0.10)

^3^SEM listed in the table were calculated for each time point or period without consideration of repeated measures. When a repeated measures design was taken into consideration, SEM were 1.3, 0.02, 0.03, and 0.01for body weight, average daily gain, average daily feed intake, and gain to feed ratio, respectively

During the period of d 1–28, ADFI of pigs fed diets containing CCDS and CWS was greater (*P* < 0.05) or tended to be higher (*P* < 0.10) compared with the control group. No difference (*P* > 0.05) in ADFI was noticed across the treatments after d 28. Overall, Treatment 2 had greater (*P* < 0.05) ADFI than the control group and tended to have higher (*P* < 0.10) ADFI than Treatments 3 and 4.

Treatment 1 had higher (*P* < 0.05) feed efficiency (ADG:ADFI) than the other three treatments from d 1 to 84. During the period of d 84–126, the feed efficiency did not differ (*P* > 0.10) between treatments. Overall, inclusion of CCDS and CWS (Treatments 2, 3, and 4) resulted in a decrease (*P* < 0.05) of feed efficiency in comparison with the corn-soybean meal based control diet.

Daily total water disappearance including water consumed with feed dry matter and nipple drinkers was 7.6, 7.1, 7.1, and 8.1 L per pig for Treatments 1 to 4, respectively, during the whole experimental period, with a tendency (*P* < 0.10) for Treatment 4 being higher than Treatments 2 and 3 (Table 4). This is mainly due to difference (*P* < 0.05) in amount of water from feed between treatments.

### Carcass characteristics

Data on carcass characteristics are presented in Table 5. Compared with the control, the other three groups had lower carcass weight (*P* < 0.05 for Treatments 2 and 3, *P* < 0.10 for Treatment 4) and backfat depth (*P* < 0.10) due to lower slaughter body weight (*P* < 0.05) in the three treatments supplemented with CCDS and CWS. Nevertheless, no differences (*P* > 0.10) in dressing percentage, muscle depth, and lean percentage were observed among the treatments.

**Table 5:** Effects of liquid feeding ethanol byproducts on carcass characteristics and belly firmness of pigs^1,2^

	Dietary treatment					
Item	Treatment 1 (corn-soybean meal)	Treatment 2 (5% CCDS + 25% CWS)	Treatment 3 (10.5% CCDS + 19.5% CWS)	Treatment 4 (graded level CCDS + graded level CWS)	SEM	*P* value
Carcass weight, kg	96.6 ^A a^	89.8 ^B^	90.3 ^B^	91.3 ^AB b^	1.5	0.012
Dressing, %	75.6	75.0	74.8	75.8	0.35	0.171
Fat depth, mm	21.9 ^a^	19.8 ^b^	19.6 ^b^	19.6 ^b^	0.6	0.030
Muscle depth, mm	67.8	65.4	65.9	65.9	0.8	0.143
Lean, %	54.2	54.3	54.4	54.4	0.2	0.849
Belly thickness, mm	34.1	32.2	33.3	31.2	2.0	0.752
Belly firmness score, degree	56.3 ^A a^	27.3 ^AB b^	24.6 ^B^	20.6 ^B^	8.5	0.021

^1^ *CCDS* corn condensed distiller’s solubles, *CWS* corn whole stillage

^2^Means within a row without common upper case letters differ (*P* < 0.05). Means within a row without common lower case letters tend to differ (0.05 < *P* < 0.10)

### Belly firmness

Data on belly firmness are shown in Table 5. A higher score indicates a firmer belly. As expected, inclusion of CCDS and CWS reduced (*P* < 0.05) or tended to reduce (*P* < 0.10) firmness of belly. There was no difference (*P* > 0.05) in belly firmness among the three groups fed diets containing CCDS and CWS.

### Sensory quality

Inclusion of CWS and CCDS did not influence (*P* > 0.10) the overall liking, flavor, tenderness and juiciness of loin chops (Table 6).

**Table 6:** Effects of liquid feeding ethanol byproducts on sensory characteristics of loins^a^

	Dietary treatment					
Item	Treatment 1 (corn-soybean meal)	Treatment 2 (5% CCDS + 25% CWS)	Treatment 3 (10.5% CCDS + 19.5% CWS)	Treatment 4 (graded level CCDS + graded level CWS)	SEM	*P* value
Overall liking score	5.72	5.80	5.72	5.56	0.17	0.531
Flavor score	5.50	5.59	5.47	5.38	0.18	0.671
Tenderness score	5.86	5.97	5.75	5.63	0.19	0.250
Juiciness score	5.40	5.54	5.25	5.15	0.19	0.153

^a^ *CCDS* corn condensed distiller’s solubles, *CWS* corn whole stillage

## Discussion

This study was designed to evaluate CCDS and CWS for feeding growing-finishing pigs. Dry matter content in CCDS (315 g/kg) and CWS (128 g/kg) used in our study was within the range of previously reported values [8, 9, 19]. However, the nutrient profile of our CCDS sample was, on a dry matter basis, quite different from the values for corn distillers solubles listed in NRC [10], with our sample having much lower ADF (7 vs. 85 g/kg), NDF (10 vs. 282 g/kg), ether extract (59 vs. 137 g/kg), calcium (0.2 vs. 3.3 g/kg), and sulfur (18 vs. 42 g/kg), but higher crude protein (258 vs. 213 g/kg), ash (132 vs. 99 g/kg), and phosphorus (24 vs. 14 g/kg), likely due to differences in ethanol production processes. During a period of 4 months, we monitored the temporal changes in nutrient composition of CCDS and CWS sampled once a month from the same ethanol plant (Guardian Energy, LLC; Janesville, MN, USA) and found that the coefficient of variation for dry matter, gross energy, crude protein, ether extract, NDF, ADF, and amino acids was 6.0, 0.8, 6.6, 44.6, 10.1, 15.4, and 10.8% (range 2.0–24.7%) for CCDS and 12.4, 0.8, 4.9, 7.9, 6.8, 17.2, and 6.0% (range 0.0–12.5%) for CWS, respectively. The temporal changes of nutrients in CCDS and CWS used in this trial might be partially related to variations of corn grain composition and/or stability of the production process over time. For example, different batches of corn might be used; the inconsistence in water inclusion rate and efficiency of distillation and centrifugation systems might also contribute to the variation. Tanghe et al. [19] analyzed five samples of condensed distiller’s solubles derived from fermentation of wheat or mixture of grains and found large variation of nutrient compositions. For example, there was 2 to 4-fold difference in contents of NDF, ADF, crude ash, and phosphorus among the five samples. The crude fat content in the five samples of condensed distiller’s solubles varied from 61 to 95 g/kg dry matter, with mean as 71 g/kg dry matter which is similar to the fat content in our CCDS sample but much lower than the fat level (190 g/kg dry matter) in CCDS reported previously [8]. Nutrient composition of our CWS sample was within the range of values reported by Han and Liu [20], who also indicated variation of amino acids contents in CWS (coefficients of variation from 1.1 to 17.5%) and distiller solubles (coefficients of variation from 8.4 to 27.3%) sampled from different plants. Compared with the CCDS sample, CWS used in this study had, on a dry matter basis, lower contents of ash, phosphorus, sulfur, but greater levels of ADF, NDF, and ether extract, and similar concentration of crude protein.

Total water disappearance (7–8 L/pig⋅d) for growing-finishing pigs in the current study was similar to the findings of Vermeer et al. [21] for pigs raised on liquid feeding systems from 22 to 115 kg. Water to feed ratio, or dry matter content in liquid diet, may affect pig growth performance [22–24] and nutrient digestibility [25, 26]. For example, Hurst et al. [24] found that feed efficiency of growing-finishing pigs was improved by 3.6% and feed intake was reduced by about 3% as the ratio of water to feed was increased from 1.5:1 to 3:1 when pigs were fed at the level of 90 to 95% of ad libitum feed intake. In the present study, dry matter content in feed was kept similar, with an attempt to remove the potential confounding effect of dry matter content.

In the current study, feeding a mixture of CCDS and CWS at a level of 30 to 50% of the dietary dry matter reduced daily gain and feed efficiency of growing-finishing pigs when compared with the corn-soybean meal-based control diet. Assuming that CWS consists of 30% of distiller’s grains and 70% of thin stillage (as-fed basis), or 80% of distiller’s grains and 20% of solubles (dry matter basis) [7], there was approximately 10, 14.5, and 14.5–24% of solubles and 20, 15.5, and 15.5–26% of distiller’s grains for Treatments 2, 3, and 4, respectively, on a dry matter basis in the current study. Research has shown that performance of growing and finishing pigs is generally not adversely impacted when including up to 30% corn DDGS [5, 27], but might be reduced in case that more than 30% corn DDGS is included [28]. However, Stein and Shurson [5] also indicated that inferior growth performance might be observed in pigs fed diets containing less than 30% DDGS due to poor quality of DDGS and/or the associated increase in dietary crude protein concentration. The crude protein level was about 1 to 3 percentage units higher in the diets containing bioethanol co-products compared with the corn-soybean meal based control diet in the current experiment. Mycotoxins in the ethanol co-products are another concern although their levels were not analyzed in the current study. Squire et al. [4] reported that feeding growing pigs 15% CCDS on a dry matter basis reduced daily gain by 6% and feed intake by 8%, and feed intake was further reduced when CCDS inclusion level was increased to 22.5% compared with the corn-soybean meal control diet.

With limited information on nutrient digestibility in CCDS and CWS, attempts were made to formulate diets containing similar levels of nutrients (ME; SID lysine, methionine plus cysteine, threonine, and tryptophan) among treatments. We measured apparent ileal and fecal digestibility of nutrients in phase 3 diets (unpublished data). Ileal digestibility of dry matter and gross energy was similar among the 4 treatments. Nevertheless, fecal digestibility of dry matter, nitrogen and energy in Treatment 4 was about 6 percentage units lower than that in the other 3 treatments, likely due to higher inclusion levels of CCDS and CWS in phase 3 of Treatment 4. Nutrient digestibility in CCDS and/or CWS might be overestimated in diet formulation of this study. We speculated that if the diets were formulated on the basis of net energy, performance of pigs fed diets containing CCDS and CWS might be improved. Nevertheless, there is a scarcity of data on net energy concentration of ethanol byproducts used in our study. Tanghe et al. [19] used the difference method to measure digestible energy (DE) in five samples of condensed distiller’s solubles derived from fermentation of wheat or mixture of grains, which varied from 3559 to 4371 kcal/kg dry matter, with average as 3989 kcal/kg dry matter. Assuming the ratio of ME to DE is 0.96, average of the ME of the five samples would be 3822 kcal/kg dry matter, which is greater than the ME value of CCDS (3415 kcal/kg dry matter) used for diet formulation in our study. The predicted net energy of the five samples ranged from 2293 to 2580 kcal/kg and averaged at 2388 kcal/kg on a dry matter basis [19], whereas the net energy of CCDS is 2436 and 2627 kcal/kg dry matter documented by NRC [11] and NRC [10], respectively. It was reported that ileal digestibility of nitrogen and most amino acids was lower in condensed distiller’s solubles than in DDGS and increasing percentage of solubles in conventional DDGS reduced ileal digestibility of amino acids in pigs [12, 29]. Furthermore, Tanghe et al. [19] found large variation of apparent fecal and ileal digestibility of energy and nutrients among five samples of condensed distiller’s solubles derived from fermentation of wheat or mixture of grains. For example, among the five condensed distiller’s solubles samples, a wide range for apparent total tract digestibility of gross energy (76–89%) and phosphorus (46–78%), and for apparent ileal digestibility of lysine (61 to 94%), methionine (75 to 86%), cysteine (50 to 75%), threonine (58 to 85%), and tryptophan (60 to 88%) was observed, indicating challenges to accurate formulation of swine diets containing ethanol co-products by using mean nutrient values of the by-products.

Dietary treatments did not affect carcass traits dramatically in this experiment when pigs were slaughtered at the same age. It was reported that including 15% of, on a dry matter basis, CCDS did not impact carcass traits (dressing, fat depth, muscle depth, lean) and loin meat quality when pigs were fed diets containing similar digestible energy and digestible amino acids and slaughtered at similar body weight [4, 8]. A recent study indicated that feeding diets containing 0, 15, 30, or 45% of DDGS and similar levels of ME and SID lysine and tryptophan to growing-finishing pigs did not have much effect on carcass leanness [6]. For carcass traits evaluation, slaughter body weight might be taken into consideration, because an increase of slaughter weight may decrease carcass leanness [30]. In the current study, slaughter body weight was about 8 kg heavier in the control group compared to the other three treatments and dressing percentage dropped numerically by 0.6 percentage unit when including 30% of ethanol by-products. Cisneros et al. [31] reported that an increase of 10 kg in slaughter live weight was associated with an increase of hot carcass weight and dressing percentage by 8.1 kg and 0.32 percentage unit, respectively. It has been suggested that high fiber diets may increase gut fill and hence intestinal mass, thus reducing dressing percentage. However, reduction of dressing percentage by the inclusion of corn DDGS is only observed in some experiments but not in other studies [5], probably partly due to differences in inclusion levels. For example, Cromwell et al. [6] reported that, at similar slaughter body weight, inclusion of up to 30% DDGS did not influence dressing percentage, but including 45% DDGS reduced dressing percentage by 0.5 percentage unit. Thirty percent DDGS may provide about 10% NDF in diet. In the current study, the CCDS and CWS samples contained, on a dry matter basis, approximately 1% and 26% NDF, respectively. If CCDS and CWS are included at levels of 20 and 30% of diet dry matter, respectively, together the two ingredients would provide 8% NDF in the diet, which is lower than the level contributed by 30% DDGS.

Reduction in belly firmness of pigs fed diets containing CCDS and CWS in the current study was most likely due to the amount and profile of fatty acids in these two ingredients. The authors are not aware of any published data regarding effects of CCDS and CWS on pork sensory characteristics. It was demonstrated that including up to 45% of corn DDGS in pig diets resulted in softer belly and an increase of iodine values, but did not affect sensory traits of loin chops [32–34]. Nevertheless, please be aware that sensory traits were evaluated with limited number of pigs in the current trial.

## Conclusion

Results from this study indicate that growth performance, carcass weight, and belly firmness were reduced in growing-finishing pigs fed diets containing high level (30–50% on a dry matter basis) of a mixture of corn condensed distiller’s solubles and whole stillage. Nevertheless, the high inclusion level of the two co-products did not adversely affect sensory traits of loin chops.
